# Stable high-level expression of factor VIII in Chinese hamster ovary cells in improved elongation factor-1 alpha-based system

**DOI:** 10.1186/s12896-017-0353-6

**Published:** 2017-03-24

**Authors:** Nadezhda A. Orlova, Sergey V. Kovnir, Alexandre G. Gabibov, Ivan I. Vorobiev

**Affiliations:** 1 Laboratory of Mammalian Cell Bioengineering, Institute of Bioengineering, Research Center of Biotechnology of the Russian Academy of Sciences, 33, bld. 2 Leninsky Ave., Moscow, 119071 Russia; 2 Laboratory of Biocatalysis, Institute of Bioorganic Chemistry of the Russian Academy of Sciences, 16/10, Miklukho-Maklaya str., Moscow, 119971 Russia

**Keywords:** CHO cells, High level expression, Stable cell line generation, Factor VIII

## Abstract

**Background:**

Recombinant factor VIII (FVIII), used for haemophilia A therapy, is one of the most challenging among the therapeutic proteins produced in heterologous expression systems. Deletion variant of FVIII, in which the entire domain B is replaced by a short linker peptide, was approved for medical use. Efficacy and safety of this FVIII deletion variant are similar to full-length FVIII preparations while the level of production in CHO cells is substantially higher.

Typical levels of productivity for CHO cell lines producing deletion variant FVIII-BDD SQ, described elsewhere, are 0.5–2 IU/ml, corresponding to the concentration of FVIII of about 0.2 μg/ml. Using standard vectors based on the cytomegalovirus promoter (CMV) and the dihydrofolate reductase cDNA we have previously obtained the cell line secreting 0.5 IU/ml of FVIII-BDD, which roughly corresponds to the previously published data.

**Results:**

An expression system based on CHO genomic sequences including CHO-EEF1A promoter and Epstein-Barr virus terminal repeat fragment allowed us to achieve 80-fold increase in the production level as compared with the conventional expression system based on the CMV promoter.

Immediately after the primary selection FVIII -producing cells secreted 5–10 IU/ml of FVIII-BDD, and after multi-stage methotrexate-driven amplification a stable clonal line 11A4H was selected, secreting 39 IU/ml of FVIII-BDD in the simple batch culturing conditions, which considerably exceeds known indicators for industrial producers of this protein. In contrast to other FVIII-BDD producing lines 11A4H accumulates low proportion of the secreted FVIII on the membrane. Its productivity may be further increased approximately two-fold by adding sodium butyrate and butylated hydroxyanisol to the culture medium.

A five-stage purification process for the factor VIII was employed. It allowed isolation of the intact FVIII-BDD as was confirmed by mass spectrometry. Purified FVIII-BDD has a specific activity of 11,000 IU/mg, similar to known recombinant FVIII drugs.

**Conclusions:**

The recombinant FVIII-BDD was produced in CHO cells without addition of any animal-derived materials, purified and characterized. Novel genetic constructions for the expression of heterologous proteins combined with optimized cultivation method allowed to obtain the secretion level of biologically active recombinant FVIII increased by almost ten times as compared with the previously published analogues.

**Electronic supplementary material:**

The online version of this article (doi:10.1186/s12896-017-0353-6) contains supplementary material, which is available to authorized users.

## Background

Blood clotting factor VIII (FVIII) is a major non-enzymatic component of the positive amplification loop in the intrinsic coagulation pathway [1]. It is present in the bloodstream mostly in the non-covalent complex with its chaperone von Willebrand factor (vWF). In the case of a blood coagulation event, FVIII undergoes specific proteolytic cleavage by the thrombin, converting it to the activated FVIII (FVIIIa) [2]. FVIIIa dissociates from vWF, binds to the surface of acidic cell membranes of activated platelets, recruits binding of the activated factor IX (FIXa) and the factor X (FX) to the ternary complex “X-ase” (or tenase), promotes specific cleavage of the FX by the FIXa to the activated FX (FXa) and allows the release of the FXa from the X-ase complex [3]. Natural human FVIII is a large glycoprotein with multiple size variants, ranging from 190 to 300 kDa. Circulating FVIII is predominantly present in the two-chain form; the heavy chain of FVIII comprises the domains A1-A2-B and the light chain the domains A3-C1-C2 [4]. Heavy and light chains of FVIII are held together non-covalently and the coordinated copper ion is considered to be crucial for maintaining the integrity of the di-chain FVIII molecule [5]. The entire B-domain of the FVIII is expendable for its hemostatic function; a significant proportion of the natural FVIII in circulation lacks the B-domain, which is removed by proteolysis [6].

Decreased level of FVIII or loss of its procoagulant function due to genetic abnormalities results in the bleeding disorder hemophilia A. Prevalence of hemophilia A is about 1:5000 men and regular infusions of natural or recombinant FVIII are the only effective treatment of this disease. Functionally active recombinant FVIII may be obtained only by secretion from cultured mammalian cells due to the complex pattern of post-translational modifications, including glycosylation and sulfation of several tyrosine residues [7], although the isolated light chain of FVIII may be expressed in methylotrophic yeasts [8]. There are two commercialized full-length recombinant FVIII products, one of them is based on BHK cells, cultured in the presence of human plasma proteins [9] and other is based on CHO cells, secreting FVIII and vWF [10]. Both these expression systems have relatively low volumetric productivity due to the low stability of the full-length FVIII even in the presence of vWF. The third variant of the commercially manufactured recombinant FVIII is the truncated protein, lacking the entire B-domain. It is secreted into the protein-free culture medium by CHO cells and does not require the presence of vWF [11]. All three preparations of recombinant FVIII are equally effective in the treatment of hemophilia A [12].

There are several factors, limiting the expression of FVIII in heterologous systems, including transcription silencers in its mRNA [13, 14], retardation in endoplasmic reticulum [15], re-adsorption of the secreted FVIII to the cell membranes [16] and its instability in the culture medium. Cell lines expressing FVIII with increased specific productivity still are of great practical importance since the cost of production of recombinant FVIII for clinical use may be significantly cut by increasing its concentration in the culture medium.

Recently we have developed a specialized plasmid vector p1.1 for stable high-level expression of heterologous proteins in CHO cells and confirmed that it enables a relatively rapid increase of cells productivity after methotrexate-driven gene amplification [17]. Here we report the use of this vector for the development of highly productive cell lines, stably secreting functionally active B-domain deleted FVIII.

## Methods

### Cloning of p.1.1-F8BDD

The DNA fragment encoding the FVIII-BDD ORF with Kozak consensus sequence was obtained by PCR using primers AD-8-AbsF (5’-ACC TCG AGG CCG CCA CCA TGG AAA TAG AGC TCT CC-3’) and AD-ONheI-R (5’-AAG CTA GCT CAG TAG AGG TCC TGT GCC-3’), the plasmid pOptivec/F8BDD [18] as a template, and Tersus polymerase mix (Evrogen, Moscow, Russia). The PCR product was subcloned into T-vector, sequenced using the primers to FVIII ORF listed in [18] and then inserted into the expression vector p1.1 (described in [17]) using *Abs*I (Sibenzyme, Novosibirsk, Russia) and *Nhe*I (Fermentas, Vilnius, Lithuania) restriction enzymes. Resulting expression plasmid p1.1-F8BDD for the transfection was purified by EndoFree Plasmid MaxiKit (Qiagen, Valencia, CA, USA) and sequence-verified.

### Cell culture

CHO DG-44 (Cat. No. A1100001, Invitrogen, Carlsbad, CA, USA) cell line, adapted to suspension culture in a chemically defined medium was used. Cells were cultured in Erlenmeyer flasks (VWR International, Radnor, PA, USA) in 30 ml of CD DG-44 medium (Invitrogen) supplemented with 8 mM L-glutamine (Invitrogen) and 0.18% of surfactant Pluronic F-68 (BASF Inc., Florham Park, NJ, USA) at 37 °C, 8% CO2, with stirring on an orbital shaker at 130 rpm. Cells were passaged every 2–3 days at the culture density 1.2 x 10^6^ live cells per ml, and diluted with fresh medium in a ratio of 1:4.

Transfection was performed using Fugene HD (Origen Biomedical, Austin, TX, USA), in a ratio of 18 μg of plasmid DNA per 1.5 × 10^7^ cells in 30 ml of culture medium. Plasmid for the transfection was linearized by the *Pvu*I or used in the supercoiled form. Transfection efficiency was determined by fluorescence microscopy 48 h post-transfection for control plasmid mixture (p1.1-F8BDD plasmid mixed 95:5 by weight with green fluorescent protein encoding plasmid pEGFP-N2). After transfection cells were cultured for 48 h without medium exchange, then transferred into selective medium ProCHO 5 (Lonza, Basel, Switzerland) and grown until cell viability was restored to 90% (15–20 days). During the cultivation in selective medium, cells were passaged every 3–5 days to the cell density 5 × 10^5^ cells/ml or less until positive growth was observed. After this, cells were passaged every 3 days at the seeding density 3 × 10^5^ cells/ml. Stably transfected cell population from the suspension culture was transferred to the 96-well plates with the culture medium Ex-Cell CHO Cloning Medium (Sigma-Aldrich, St. Louis, MO, USA), supplemented by the 8 mM of L-glutamine and 50–400 nM MTX. The growth of colonies was monitored at the 10th and 14th days of cultivation. Growth-positive wells were screened by ELISA, culture medium in antigen-positive wells was changed and colonies were grown close to the confluence. Alternatively, at 48 h post-transfection cells were seeded into 96-well plates at a density of 10,000 cells per well, 200 μl per well of AOF Ex-Cell CHO Cloning Media (Sigma-Aldrich) medium with 50 nM MTX and 8 mM of L-glutamine at 37 °C, 5% CO2. Cells were maintained as described above.

Cells from most productive colonies obtained by both techniques were transferred to 48-well plates, cultivated for 5 d and screened again by ELISA. Best secreting oligoclonal lines were further expanded and readapted to suspension growth in ProCHO 5 medium with 8 mM L-glutamine during three sequential passages in 24-, 12- and 6-well plates.

FVIII concentration in the culture medium was measured 48 h post-transfection and at the end of the selection for stable transfectants. Oligoclonal cell line with the highest level of secreted FVIII, obtained in the presence of 50 nM MTX, was used for further amplification of bicistronic FVIII-dhfr cassette. It was grown in the ProCHO 5 medium with 8 mM of L-glutamine in the presence of increasing concentrations of methotrexate (MTX). Each amplification step lasted for 14–25 days until cell viability restored to 90%. Concentration of secreted FVIII was measured by ELISA and/or clotting test at the end of each step. Transgene-amplified oligoclonal cell line, obtained by the highest MTX selection pressure possible (4 μM) was cloned by limiting dilution in AOF Ex-Cell CHO Cloning Media (Sigma-Aldrich) with 8 mM alanyl-glutamine (Invitrogen) and HT (hypoxanthine/thymidine) supplement (Invitrogen), without MTX. Cells were seeded into 96-well plates at a density of 0.5 cells per well, 200 μl of medium per well at 37 °C, 5% CO2. The growth of single colonies was monitored at the 10th and 14th days of cultivation. The colonies were screened by ELISA, culture medium in positive wells was changed and colonies were grown close to the confluence. According to the results of the subsequent ELISA of actively growing colonies the most productive of them were transferred to 48-well plates. Clonal lines with the best levels of secretion were further expanded and readapted to suspension growth in ProCHO 5 medium with 8 mM L-glutamine during three sequential passages in 24-, 12- and 6-well plates.

Conditioned medium from 6-well plates was analyzed by ELISA. The top three cell lines were moved to Erlenmeyer flasks and evaluated by growth rate and specific productivity. Preparative batch cultivation was conducted in 500 ml Erlenmeyer flasks, 125 ml of culture medium per flask. Cells were seeded at a concentration of 2.5 × 10^5^ cells/ml and cultured without medium change to a density of 3 × 10^6^ cells/ml (4–5 days).

### ELISA

Concentration of the factor VIII antigen (FVIII:Ag) was measured by ELISA using polyclonal antibodies to FVIII (LifeSpan BioSciences, Seattle, WA, USA) at 50 ng per well and specific MAb A2 to FVIII heavy chain described in [18]. Serial dilutions of normal calibrated human plasma (NPO Renam, Moscow, Russia) in PBS with 1% bovine serum albumin (BSA) were used as a standard. Samples were also diluted in PBS with 1% BSA.

### Clotting assays

One stage clotting assay with chromogenic substrate was performed by the TECHNOCHROM® F VIII:C kit (Technoclone GmbH, Vienna, Austria); two stage clotting assay was performed by the optical coagulation analyzer ThromboScreen 400c (ThermoFisher Scientific, USA) and the Factor VIII- test reagent kit (NPO Renam), using normal calibrated human plasma (NPO Renam) as the initial standard and one stage assay-characterized sample of the FVIII-BDD preparation as the working standard. Specific activity of several FVIII-BDD samples was 1.7 times lower in the two-stage assay than in the one-stage assay if human plasma was used as the activity standard. Samples were diluted with the imidazole buffer with 1% BSA (NPO Renam).

### Medium modifications

Effect of alkanoic acids in the culture medium on FVIII secretion was assessed in batch cultures, seeded as 3.75 × 10^5^ cells/ml and cultured for 3 days unless stated otherwise. Sodium butyrate and sodium propionate were dissolved in water at 1 M and adjusted to the pH 7.2 by HCl prior to addition to the culture medium. Antioxidant butylated hydroxyanisole was dissolved in DMSO at 2.5 M prior to addition to the culture medium, final concentration 0.1 mM; addition of dichlorofluorescein (DCF, Invitrogen) and subsequent flow cytometry analysis for oxidative stress level was performed as described in [19].

### Oxidative stress induction and flow cytometry

Cells of the 11A4H line were seeded as 3.5 × 10^5^ cells/ml in the protein-free ProCHO-5 medium and cultivated for 4 days in the shake flask. On 3^rd^ and 4^th^ day 1 × 10^6^ cells were sampled. Then samples were stained with 5 μM of CM-H2-DCF-DA (Invitrogen) for 20 min on ice and counterstained with 1.5 μM propidium iodide just before flow cytometry analysis on Cytomics FC500 instrument (Beckman Coulter). Direct reads were gated on FS/SS scatter against intact control cells sample and then subsequently gated for propidium iodide exclusion (channel FL-3 620 nm). Generation of ROS were assessed as green fluorescence of activated DCF by measuring in FL-1 (525 nm) channel for 10^5^ gated cell reads, every obtained FL-1 scatter histogram were unimodular, mean values were calculated for core 5 ml of intact cell culture with H_2_0_2_ in final concentrations 10 μM, 100 μM and 1000 μM directly prior to live staining.

### Quantitative PCR, PCR, RT-PCR

Copy number of the expression cassettes in the genome was determined by the quantitative real-time-PCR (qPCR). A calibration curve was prepared using serial dilutions of highly purified p1.1-F8BDD plasmid. Genomic DNA was isolated by Wizard SV Genomic DNA Purification System (Promega, USA) and quantified by the Qubit Fluorometer (Invitrogen), using dsDNA HS kit (Invitrogen) and external DNA concentration standard, prepared in-house from the highly purified plasmid DNA, quantified by UV spectrophotometry. Concentration of the genomic DNA was validated by control qPCR with primers to PPIB gene region, presumably unique to the CHO cells genome according to the BLAST search results. Samples of genomic DNA with the determined copy numbers of PPIB less than 0.3 per haploid genome or more than 2.0 were discarded. Weight of one CHO haploid genome was established as 3 pg according to [20].

The mRNA levels were assayed by reverse transcription and quantitative PCR (RT-qPCR). Total RNA was isolated by the RNeasy Mini Kit (Qiagen), its concentration was determined by UV spectroscopy, purification quality was confirmed by monitoring the A260/A280 ratio (1.8–2.0); integrity was verified by electrophoresis in 1% agarose gel. The cDNA was synthesized with the reagent kit Mint (Evrogen) using 1 μg of total RNA per sample. The ΔΔCq method [21] was used to calculate mRNA expression relative to β-actin.

Primers (listed in Additional file 1: Table S1) were designed by the Beacon Designer v7.51 program (PREMIER Biosoft International, Palo Alto, CA). The unique primers allowing selective amplification of the expression cassette were chosen for the genome copy number analysis, and primers located in the different exons or at the exon junctions were used for mRNA expression level analysis.

QPCR was performed using qPCRmix-HS SYBR reaction mixture (Evrogen) and iCycler iQ thermocycler (Bio-Rad, USA). Calculations of threshold cycles, calibration curves, PCR efficiency and copy numbers were made by the iCycler Iq4 program. All determinations were repeated 3 times, in 3–5 replicates, sample volume 25 μl.

PCR analysis of the whole FVIII ORF area was performed for genomic DNA and cDNA in the essentially same conditions. Templates were taken as 10 ng per tube, reaction volume was 10 μl. Primers AD-8-AbsF and AD-ONheI-R, described in the *Cloning* subsection, were used; Encyclo PCR kit (Evrogen) was employed. Temperature gradient was from 53 °C to 68 °C. Amplification program was 3’ at 95 °C; 25 cycles as 15” at 95 °C, 53–68 °C for 15”, 72 °C for 3’. Final elongation was 72 °C for 5’. Amplification products were electrophoresed on 0.8% agarose gels and stained with ethidium bromide.

### Southern blot hybridization

Biotinylated probes for Southern blotting were prepared by the Biotin DecaLabel DNA Labeling Kit (Fermentas). Template for the probes was pAL-ID plasmid (containing regions present in expression plasmids p1.1 including origin of replication, a beta-lactamase gene, EMCV IRES, DHFR ORF [17]) or a PCR product corresponding to the fragment of FVIII-BDD ORF. Genomic DNA was digested with *EcoR*I (Fermentas) for 16 h, precipitated by ethanol, separated by 0.8% agarose gel. Gel transfer to an Amersham Hybond-N+ membrane (GE Healthcare, USA) was performed according to the manufacturer’s protocol in 20x SSC buffer (3 M NaCl, 0.3 M Na_3_C_6_H_5_O_7_) for 16 h. DNA was fixed by heating the dried membranes at 80 °C for 2 h. Prehybridization and hybridization were conducted according to [22] in the buffer containing 7% SDS, 0.5 M Na_2_PO_4_, pH 7.2, 1% BSA for 16 h at 65 °C. Membrane was washed according to the manufacturer’s protocol and stained by Biotin Chromogenic Detection Kit (Fermentas).

### SDS-PAGE and immunoblotting

Samples of conditioned medium were clarified by centrifugation and concentrated 30 times by precipitation with trichloroacetic acid. Cell lysates were prepared using a modified RIPA buffer (50 mM Tris -HCl, pH 7.4, 1% NP- 40, 0.25% sodium deoxycholate, 150 mM NaCl, 1 mM Na-EDTA) with protease inhibitor cocktail (Sigma-Aldrich) and normalized to total protein concentration, determined by QuantiPro ™ BCA Assay Kit (Sigma-Aldrich). Samples were separated by electrophoresis in 7.5% denaturing polyacrylamide gel, sample load was 10 μg per well for cell lysates and the equivalent of 0.5 ml of conditioned medium. Gel slabs were stained by colloidal Coomassie stain (Fermentas) according to manufacturer's procedure or used for blotting. Transfer to the membrane, blocking, hybridization and staining were performed as described previously [18].

### Purification of FVIII

Purification of FVIII from the cell culture supernatant was carried out as described in [23] with changes.

All solutions used for the purification, except the final composition, contained 0.02% Tween-80. To the conditioned culture medium or cell suspension, NaCl was added to 0.3 M and CaCl_2_ to 27 mM. The resulting suspension was incubated for 30 min with stirring at 37 °C, clarified by centrifugation, supplemented with 10 mM of L-histidine pH 7.0 and 0.02% of Tween-80 and applied in 100 ml portions to the 1 ml HiTrap Capto MMC column (GE Healthcare), equilibrated with a solution 1-A (0.3 M NaCl, 10 mM CaCl2, 10 mM L-histidine, pH 7.0), flow rate 1 ml/min.

Column was washed by 10 volumes of solutions 1-B, 1-C and 1-D at 2 ml/min. Solution 1-B contained 1 M NaCl, 50 mM CaCl_2_, 50 mM L- histidine, pH 6.5. Solution 1-C contained 0.1 M NaCl, 50 mM CaCl_2_, 50 mM L-histidine, pH 6.5. Solution 1-D contained 0.3 M NaCl, 10 mM CaCl_2_, 10 mM L-histidine, 0.25 M L-arginine-HCl, 10% ethylene glycol, pH 6.5. FVIII was eluted at 1 ml/min by the solution 1-E (0.3 M NaCl, 20 mM CaCl_2_, 20 mM L- histidine, 0.8 M L- arginine-HCl, 50% ethylene glycol, pH 6.5).

Eluted FVIII solution was diluted 8-fold with a solution 2-A (10 mM NaCl, 10 mM L-histidine, 10 mM CaCl_2_, pH 6.5) and applied to the 1 ml Tricorn 5/50 column (GE Healthcare) with the SP Sepharose FF (GE Healthcare), equilibrated with a solution 2-B (0.15 M NaCl, 10 mM L- histidine, 10 mM CaCl_2_, pH 6.5), flow rate 2 ml/min. Column was washed with 20 ml of the solution 2-B and FVIII was eluted by the solution 2-C (0.34 M NaCl, 35 mM CaCl_2_, 45 mM L-arginine-HCl, 0.2 M sorbitol, 10 mM L-histidine, pH 6.5). Resulting solution of the semi-purified FVIII was immediately frozen in liquid N_2_ and stored in the −80 °C freezer.

Thawed FVIII solution was applied on the 1 ml Tricorn 5/50 column, filled with the immunoaffinity resin VIII Select (GE Healthcare) at 0.2 ml/min. Prior to the application of the sample, the column was equilibrated with the solution 3-A (0.3 M NaCl, 20 mM CaCl_2_, 20 mM L- Histidine, pH 6.5). The column was washed sequentially at a rate of 2 ml/min by 10 ml of the solution 3-A and 3 ml of the solution 3-B (1 M NaCl, 20 mM CaCl_2_, 20 mM L-Histidine, pH 6.5). FVIII was eluted with the solution 3-C (1.5 M NaCl, 20 mM CaCl_2_, 20 mM L- Histidine, 50% ethylene glycol, pH 6.5).

Eluate was diluted 15-fold with the solution 4-A (10 mM NaCl, 10 mM L- histidine, 10 mM CaCl_2_, pH 6.5) and applied at 2 ml/min to the 1 ml HiTrap Capto Q column (GE Healthcare), equilibrated with the solution 4-B (0.1 M NaCl, 0.02 M L-histidine, 20 mM CaCl_2_, pH 6.5). Column was washed with 20 ml of the 4-C solution (0.3 M NaCl, 20 mM L-histidine, 20 mM CaCl_2_, pH 6.5). Elution was performed by the 4-D solution (0.4 M NaCl, 0.02 M CaCl_2_, 20 mM L-histidine, pH 6.5).

FVIII solution was applied in 2 ml portions at 0.4 ml/min flow rate to the size exclusion column Tricorn 10/300 Superdex 200 (GE Healthcare), equilibrated with the 5-A solution(9 g/L NaCl, 0.25 g/L CaCl_2_, 1.5 g/L L-histidine, 0.01% Tween-80, 3 g/L sucrose, pH 7.0) at the flow rate 1 ml/min. Monomer of the FVIII in the long-term storage solution was collected in the final volume of 5–8 ml, divided into small aliquots, frozen in liquid N_2_ and stored frozen for further analysis.

### Mass spectrometry

Coomassie-stained bands of FVIII chains were cut from the polyacrylamide gel, washed twice (to remove the dye) with 100 μl of 40% acetonitrile in 0.1 M NH_4_HCO_3_ for 20 min at 37 °C, dehydrated by 100 μl of acetonitrile and completely dried *in vacuo*. Four μl of 15 μg/ml modified trypsin solution (Promega) in 50 mM NH_4_HCO_3_ added to each 3–4 mm^3^ gel piece. Hydrolysis was carried out for 5 h at 37 °C, then 7 μl of 0.5% trifluoroacetic acid (TFA) in 10% aqueous acetonitrile solution was added and mixed thoroughly. The upper layer of the solution above the gel was used for MALDI-TOF analysis.

2 μl aliquots of the peptide mixtures were mixed with 0.5 μl of 2.5-dihydroxybenzoic acid solution (Aldrich, 20 mg/ml in 20% aqueous acetonitrile, 0.5% TFA), applied to target spots and air dried.

Mass spectra were obtained on a MALDI-TOF instrument UltrafleXtreme (BrukerDaltonics, Germany) in the positive ion mode with the reflectron. For each sample analyzed the accuracy of the monoisotopic mass measurements was maintained as at least 0.003% (30 rrm) by the calibration on trypsin autolysis peaks. Spectra were obtained in the mass range 700–4500 m/z. Peptide mapping was conducted using the GPMaw program (Lighthouse data, Denmark).

## Results

### Establishment and characterization of BDD-FVIII producing cell line

**Fig. 1:**
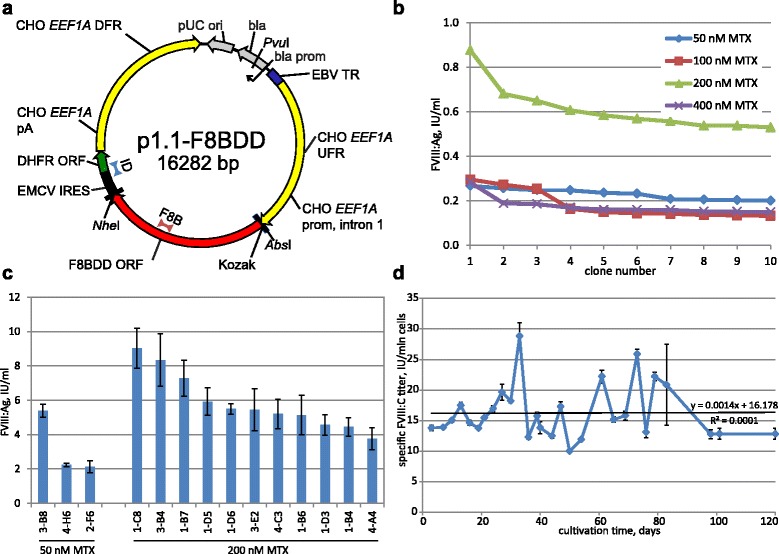
Expression plasmid map, productivity of primary oligoclonal cell lines and long-term secretion rate dynamics of the selected clonal line. Panel **a** — map of the expression plasmid, CHO EEF1A1 DFR – downstream flanking area of the EEF1A1 gene, UFR – upstream flanking area; EBV TTR – fragment of the long terminal repeat from the Epstein-Barr virus; pUC ori – replication origin; bla, bla prom – ampicillin resistance gene and the corresponding promoter; CHO EEF1A1 prom, intr1 – promoter and the first intron of the EEF1A1 gene; F8BDD ORF – open reading frame of the FVIII gene with the BDD deletion; pA – polyadenylation signal. Positions of qPCR amplicons “F8B” and “ID” are marked by *red* and *blue triangles*, amplicon lengths are not to scale. Panel **b** – culture medium samples taken from 96-well plates, testing was done when first 10% of the wells reached confluence; panel **c** – selected cell lines readapted to suspension culture and tested at 1.2 × 10^6^ cells/ml. Panel **d** - periodic culturing of the 11A4H cell line. Cells were passaged every third day, FVIII:C titer was measured after 3 days of culture. Stated concentrations of the MTX were used in the corresponding selection of cell cultures. Results are means, *error bars* represent standard deviation, *n* = 2. Raw data for figures are presented in corresponding Additional file 3

The expression construct p1.1-F8-BDD [GenBank: KY682701] was obtained by ligating the FVIII-BDD SQ ORF preceded by the Kozak consensus sequence into expression vector p1.1. Natural FVIII UTRs were completely removed; therefore in the resulting expression construct, FVIII ORF was entirely relocated into the context of *EEF1A1* gene (Fig. 1a).

The FVIII:Ag level for the CHO DG-44 cells transiently transfected with this plasmid in the supercoiled form was 9 mIU/ml. The observed low level of FVIII:Ag in the transiently transfected cells corresponds to the low transfection efficiency, not exceeding 3% for the 16 kbp plasmid used. Meanwhile, CHO DG-44 cells stably transfected with the CMV promoter-based plasmid pOptivec/F8BDD displayed 2-fold lower FVIII:Ag level under the same growth conditions [18] despite higher transfection efficiency. Transfection of cells by the linearized expression plasmid p1.1-F8-BDD gave no detectable product 48 h post-transfection and no stably transfected cells after 3 weeks of selection.

Two selection strategies were used for transfected cells (cell lines generation scheme is depicted in the Additional file 2: Figure S1). Generation of a stably transfected population in suspension culture in the absence of both MTX and HT with subsequent selection of oligoclonal lines in adherent culture or direct selection of stably transfected oligoclonal lines in adherent culture immediately after transfection.

Stably transfected population (with FVIII:Ag level 134 mIU/ml) was plated into 96-well plates in the presence of 50–400 nM MTX. Multiple FVIII:Ag-positive colonies were observed in all cases (data not shown). It should be noted that most of wells in the plates with 50–200 nM MTX in the culture medium gave multiple colonies, but in the case of 400 nM MTX supplementation the proportion of wells with growing colonies dropped to less than 10% from total. Subsequently, the ten most productive wells in the plates with 50, 100 and 200 nM MTX were chosen from 200–300 wells with actively growing colonies and the 10 most productive wells for plates with 400 nM MTX supplementation were chosen from less than 40 wells with colonies. In these conditions, 200 nM MTX supplementation resulted in the sharp increase of the maximal titer in first 10 most productive wells (Fig. 1b).

For direct selection transiently transfected cells were seeded 48 h post-transfection into 96-well plates in the presence of 50 nM MTX and grown in the same manner.

Cells from the most productive wells, obtained by selection and subsequent amplification in the presence of 200 nM MTX or by direct selection with 50 nM MTX, were expanded and re-adapted to suspension culture. Levels of FVIII:Ag in the culture medium (Fig. 1c) ranged from 5 to 10 IU/ml which is comparable to typical published values for clonal cell lines producing FVIII-BDD [24]. Thus one-step transgene amplification under adherent conditions and direct selection of transiently transfected cells in the presence of 50 nM MTX resulted in oligoclonal cell lines, secreting FVIII-BDD in the range of 2–8 IU/ml.

Oligoclonal line 3-B8, obtained by the direct selection in the presence of 50 nM MTX and secreting mostly the intact two-chain form of FVIII-BDD and virtually no degraded FVIII chains, according to Western blot data (data not shown), was selected for further amplification in the suspension culture.

Significant decrease of cell viability and inhibition of cell growth rate were detected for the 3-B8 cell line at the MTX level 1 μM. After gradual elevation of MTX concentration to 8 μM, cell viability above 85% was not achieved after 20 days of cultivation and maximal sustainable level of MTX was 4 μM. No significant change in the FVIII-BDD concentration was detected while increasing MTX from 1 μM to 4 μM (Table 1), but we suggested that higher level of the MTX may give rise to cell clones with higher specific productivity. For this reason we choose the oligoclonal cell line obtained in the presence of 4 μM MTX for clonal cell lines generation by limiting dilution. Cells were plated into twenty 96-well culture plates in the absence of MTX and in the presence of HT, resulting in 361 single colonies. The most productive colonies were expanded and re-adapted to suspension culture conditions.

**Table 1:** Cell lines, secreting FVIII

Cell line name	Steps of selection and target gene amplification by MTX	FVIII concentration, IU/ml	Specific productivity, μIU/cell/day
3-B8	50 nM	5.4 ± 0.4^a^	-
3-B8 1 K	50 nM → 500 nM → 1 μM	20.1^a^	-
3-B8 2 K	50 nM → 500 nM → 2 μM	18.8^a^	-
3-B8 1 K 2 K	50 nM → 500 nM → 1 μM → 2 μM	17.6	-
3-B8 1 K 4 K	50 nM → 500 nM → 1 μM → 4 μM	18.6	-
13B4F	50 nM → 500 nM → 1 μM → 2 μM → cloning	39.0 ± 5.5	11.8
11A4H	50 nM → 500 nM → 1 μM → 2 μM → cloning	39.4 ± 3.4	14.2
21A7B	50 nM → 500 nM → 1 μM → 2 μM → cloning	34.4 ± 0.8	11.8

^a^- FVIII concentration determined by ELISA

Three lead cell lines were selected and further expanded to shake flask cultures. The best clonal line 11A4H had specific productivity of 14.2 μIU/cell/d and acceptable doubling time of 28.5 h. Batch culture of 11A4H in shake flask, seeded at 3 × 10^5^ cells/ml in the protein-free ProCHO medium gave a maximal concentration of FVIII:C 39 ± 3 IU/ml at day 4, with final viable cell count (VCD) of 3.5 × 10^6^ cells/ml and viability over 85%. Further culturing without medium change resulted in sharp drop in FVIII:C level and subsequent drop in cell concentration and viability (data not shown).

Perfusion-like culturing was performed by seeding cells in fresh culture medium at 1.5 × 10^6^ cells/ml and changing medium daily by centrifugation and subsequent resuspension of the cell pellet in the fresh medium. In these conditions, FVIII:C level was in the range of 40–60 IU/ml for several days. We suggest that the observed increase of FVIII:C level in perfusion-like culture is due to the shorter time the secreted protein remained in culture medium exposed to proteases.

Since MTX was removed from the culture media at the stages of cell cloning and expansion, we expected that the resulting clonal cell line would maintain a high level of FVIII secretion for a sufficiently long time needed for the creation of cell banks and subsequent industrial manufacturing of the FVIII protein. For the 11A4H cell line the observed specific level of FVIII secretion (ratio of FVIII:C and VCD) showed no tendency to decrease during 120 days of cultivation (at least 70 generations) in the absence of MTX (Fig. 1d).

**Fig. 2:**
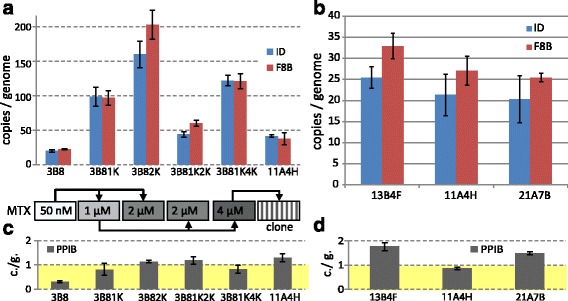
qPCR analysis of the expression cassette copy numbers per haploid genome. Panel **a** - average copy number increase during MTX-driven amplification in oligoclonal cell lines. Panel **b** - expression cassette copy number per haploid genome for clonal lines 13B4F, 11A4H, 21A7B. Panels **c**, **d** - copy numbers for the control sequence (PPIB), supposedly unique to the CHO genome. ID- primers toward IRES-DHFR region, F8B - primers toward FVIII-BDD ORF. Positions of the amplicons on the plasmid are depicted on Fig. 1a. One representative experiment from 3 is shown. *Error bars* represent standard deviation, *n* = 3

The transgene copy number in the genome of the cell lines expressing FVIII was determined by qPCR. Two transgene-specific areas of the integrated genetic cassette were used for analysis — SQ linker region of the FVIII ORF and the junction area between EMCV IRES and the DHFR ORF. Endogenous PPIB gene region, presumably unique in the CHO cells genome, was used as a single-copy control amplicon. For oligoclonal cell populations elevation of the transgene copy number during the first step of the MTX-driven transgene amplification and subsequent changes of this number in both directions are seen (Fig. 2a and c), but the observed FVIII:C levels remain mostly unchanged (Table 1). The transgene copy number for the lead clonal cell lines was not proportional to their specific productivity. Moreover, the transgene copy number for all three lines was strongly reduced compared to the parent oligoclonal line (Fig. 2b and d). Apparently, a significant portion of genomic inserts that occurred during the MTX-driven amplification was unstable or transcriptionally inactive. It should be noted that the copy number of the expression cassette in the genome of best-producing cell line 11A4H is moderate, only about 30 copies per haploid genome. While for CMV promoter-based producer cell lines, hundreds or even thousands of target gene copies per haploid genome were described elsewhere [25].

**Fig. 3:**
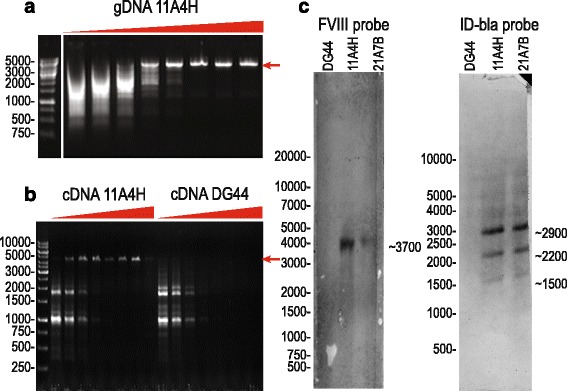
Integrity of the FVIII’s ORF in the genome DNA and mRNA of the 11A4H cell line and Southern blot analysis of genomic DNA. Panel **a** – PCR with the annealing temperature gradient for genomic DNA from 11A4H cell line. Panel **b** – PCR for cDNA, prepared from 11A4H cell line and control un-transfected CHO DG44 cells. Panel **c** – Southern blot analysis of genomic DNA from 11A4H and 21A7B cell lines and from un-transfected control. “FVIII probe” - probe toward FVIII ORF, “IRES-DHFR-bla” – probe toward corresponding expression vector areas. Contrast for scans of developed membranes was enhanced to add visibility. *Red triangles* depict annealing temperature gradients of 53 °C–68 °C for all reactions, DNA fragments sizes in bp. Expected size of the correct PCR product – 4818 bp. Images of the agarose gels used for blotting are presented on the Additional file 4: Figure S3

To ensure that genomic copies of inserted expression cassette in the candidate cell line 11A4H did not undergo large rearrangements (deletions) during the MTX-driven gene amplification process the genomic DNA isolated from A11H4 line was analyzed by PCR amplification of the whole FVIII ORF region using temperature gradient. The single PCR product corresponding to the full-length ORF (4818 bp) was observed, no truncated forms were detected. So we could conclude that all genome-integrated copies of the expression cassette contain the intact FVIII-BDD ORF without large deletions. (Fig. 3a). PCR product was also cloned and three plasmid clones were subjected to complete sequencing of the insert. All clones contained correct ORF of the FVIII without mutations, so we have assured that 11A4H cell lines codes only for correct FVIII protein (data not shown). Absence of incorrectly spliced or truncated FVIII mRNA was confirmed by PCR of total cDNA with temperature gradient, also showing single specific product of the right size (Fig. 3b).

Consistency of the genetic inserts in the genome of the candidate cell line 11A4H and the backup cell line 21A7B was investigated by Southern blotting using the FVIII-BDD ORF-specific probe (Fig. 3c) and another probe specific for the IRES, DHFR and the area of the *bla* gene (Fig. 3d). Single hybridization band of the correct size (app. 3700 bp) was observed for the FVIII-BDD ORF probe, indicating that both cell lines contain no truncated sequences of the target gene. In the case of IRES, DHFR, bla probe, three hybridization bands were visible. Two of them correspond to the predicted size of the restriction fragments, containing the IRES-DHFR area of the genetic cassette (app. 2200 bp) and the complete “bacterial maintenance” region of p1.1-F8BDD plasmid (2900 bp). Thus IRES-DHFR region apparently remains connected to the FVIII-BDD ORF sequence; this conclusion is confirmed by the qPCR data on copy numbers for FVIII and IRES-DHFR amplicons.

Third hybridization band had the size of app. 1500 bp and could correspond to the junction area between the “bacterial maintenance” region of the plasmid and the host cell DNA or to the junction between tandem copies of the genetic insert. We can assume that IRES-DHFR area of the p1.1-F8BDD plasmid remained intact in the genome of the A11A4H cell line and the non-functional “bacterial maintenance” region of the plasmid was present in both intact and truncated form. Our data cannot rule out the presence of unexpected genome integration points in the areas of the EEF1A1 UFR and EEF1A1 DFR parts of the plasmid. Due to the presence of the same DNA sequences in the genome of untransfected host cells, the genome insertion events of the EEF1A1 UFR and EEF1A1 DFR parts of the plasmid cannot be confirmed by Southern blotting and will be additionally studied by the new generation sequencing methods.

**Fig. 4:**
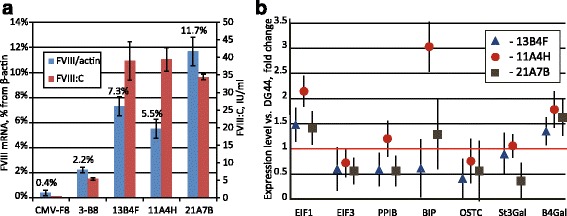
qPCR analysis of FVIII mRNA and expression levels of various housekeeping genes for clonal FVIII-secreting cell lines. Panel **a** – levels of FVIII mRNA relative to β-actin mRNA. Panel **b** - expression levels of genes involved in protein synthesis and processing compared to the parent cell line CHO DG-44. Sample “CMV-F8” on the panel **a** – clonal cell line DG-BDDFVIII-18, described in [18]. Data on the panel **b** normalized to β-actin. EiF1a1- eukaryotic translation initiation factor 1a, EiF3- eukaryotic initiation factor 3, PPIB- peptidyl-propyl isomerase B, BIP –immunoglobulin-binding protein (BiP, Grp78); OSTC- oligosaccharyltransferase complex subunit; St3gal – ST3 beta-galactoside alpha-2,3-sialyltransferase 3; B4gal - beta-1,4-galactosyltransferase 1. FIXp – control line producing factor IX. One representative experiment from 3 is shown. *Error bars* represent standard deviation, *n* = 3-4

Relative levels of FVIII mRNA for three lead cell lines (Fig. 4a) were not proportional to their specific productivities, indicating that actual secretion level of FVIII is not simply limited by the transcribed mRNA levels. At the same time, relative level of FVIII mRNA in the parental line 3-B8 is at least 2.5 times lower, than in the clonal lines and the end-titer of FVIII for this line is approximately 6 times lower. Control clonal cell line DG-BDDFVIII-18 [18], expressing essentially the same FVIII molecule, coded by the CMV promoter-based plasmid, has even lower level of FVIII mRNA and lower secreted FVIII titer. Thus relatively high level of FVIII mRNA is required for high secretion rate, although quantitative levels of FVIII mRNA and the secreted protein are not proportional.

It is known, that high levels of translation of FVIII mRNA results in the retention of the immature FVIII pro-protein in the ER and drives the activation of the UPR (unfolded protein response) [26]. UPR is coupled to oxidative stress induction and increased cell death rate. These effects are much more pronounced in the case of full-length FVIII overexpression, although they are still detectable in the case of FVIII-BDD [26]. Common marker of the UPR activation is the up-regulation of the BiP chaperone, which directly interacts with the FVIII pro-protein in the ER. In two of the three lead cell lines obtained by us levels of BiP mRNA were found to be similar to the baseline (Fig. 4b). In the case of the most productive cell line 11A4H, level of BiP expression was increased approximately threefold. Similar increase of the BiP level was described for the BHK cells, secreting human FVIII at the 2–3 IU/10^6^ cells/d rate [26].

### Modifications of the culture medium

The secretion level of several recombinant proteins can be increased by adding sodium butyrate to the culture medium [27]. Expression level of FVIII is also boosted by sodium butyrate [28], probably due to inactivation of transcription silencer, present in the mRNA of FVIII [29]. A similar effect was described for sodium propionate addition [24]. At the same time, addition of sodium butyrate to the culture medium induces endoplasmic reticulum stress in the FVIII-producing cells, as was shown in [19] and damages the cells. Major consequence of endoplasmic reticulum stress induction is the oxidative stress, which can be diminished by the membrane-permeable antioxidant butylated hydroxyanisole (BHA).

**Fig. 5:**
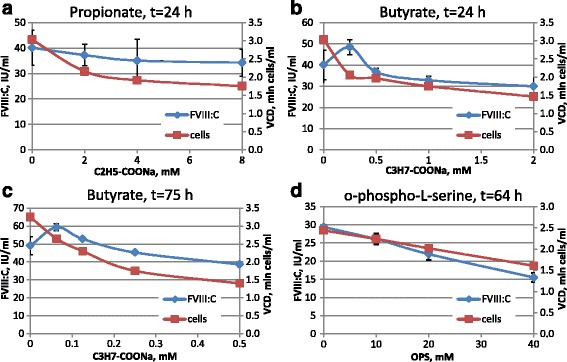
Effects of sodium propionate, sodium butyrate and o-phospho-L-serine on FVIII:C level and cell growth of the 11A4H cell line. Growth time is stated on corresponding panels. Cultures, presented on panels **a**, **b**, **d** were seeded at the VCD 1.4 × 10^6^ cells/ml; panel **c** - 3.75 × 10^5^ cells/ml. *Error bars* indicate standard deviations, *n* = 2

**Fig. 6:**
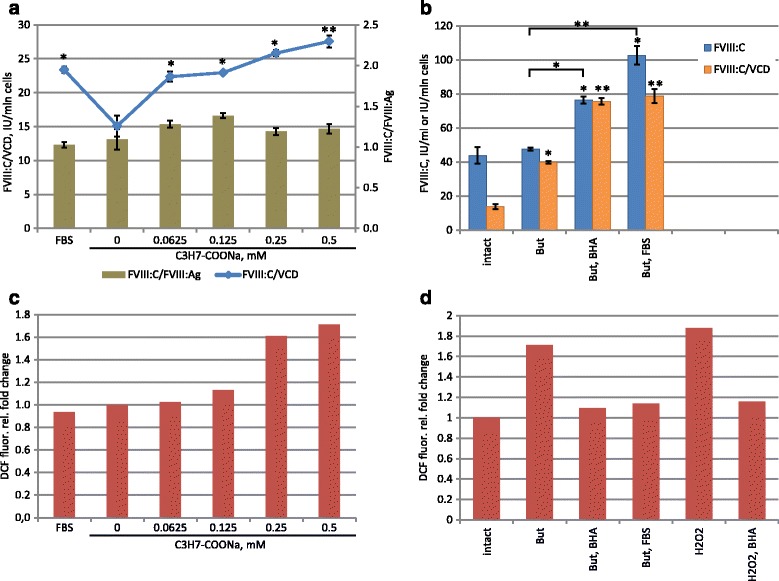
Induction of oxidative stress by the sodium butyrate and its prevention by the BHA or fetal bovine serum increases volumetric productivity of the 11A4H cell line in batch culture. Panels **a**, **c** – batch cultivation in the presence of sodium butyrate, 72 h. Panels **b**, **d** – batch cultivation in the presence of 0.5 mM sodium butyrate (But), 10 μM BHA (BHA), 10% FBS (FBS), 0.1 mM H_2_O_2_ (H_2_O_2_), 96 h. Panels **c**, **d** – flow cytometry analysis of DCF-stained cells, samples match those on panels **a**, **b**. Cells were seeded at 3.75 × 10^5^ cells/ml. Data are presented as mean ± standard deviations, *n* = 2–3. The statistical significance of the difference between groups was calculated by the unpaired t-test (* - *P* < 0.05; ** - *P* < 0.01). The difference was assessed between the “intact” or “0 mM” group and other groups, if not indicated by brackets

We applied sodium butyrate and sodium propionate supplements to batch cultures of 11A4H cells for 24 h. In the case of sodium propionate treatment both FVIII:C and VCD were decreased (Fig. 5a), but sodium butyrate, used at 0.25 mM, allowed the increase of FVIII:C and simultaneous decrease of VCD (Fig. 5b). Addition of up to 0.5 mM of sodium butyrate allowed cell proliferation, so 20% increase of volumetric productivity was recorded for batch culture in the presence of 62.5 μM of sodium butyrate (Fig. 5c) after 3 days of cultivation. Ratio of procoagulant activity of secreted FVIII and its concentration as ELISA antigen (FVIII:C/FVIII:Ag) was not affected significantly by the addition of sodium butyrate (Fig. 6a) up to 0.5 mM, indicating that sodium butyrate does not damage the specific activity of the FVIII.

We measured levels of reactive oxygen species (ROS) generation as a common marker of the oxidative stress and found that the ROS level is directly proportional to the concentration of sodium butyrate (Fig. 6c). The addition of 10 μM BHA or 10% of fetal bovine serum was sufficient to reduce the ROS generation level to baseline values (Fig. 6d). At the same time, both specific productivity and volumetric productivity in the 96 h batch culture of 11A4H cells were significantly increased (Fig. 6b). Thus the volumetric productivity of the 11A4H cell line in the batch culture can be almost doubled by using non-toxic and inexpensive medium supplements.

### Membrane-bound fraction of the secreted FVIII

Significant fraction of the secreted FVIII binds to the cell membrane, preferably to the acidic phosphatidylserine-containing membranes [30]. In the case of full-length FVIII, membrane-bound fraction was estimated as ¾ of all extracellular FVIII [16] and in the case of FVIII-BDD it may increase up to 90% [31]. Membrane-bound FVIII-BDD may be liberated during the cultivation by the addition of vWF, annexin V or water-soluble phosphatidylserine analogue — o-phospo-L-serine [32]. Membrane-bound FVIII also may be dissociated from the cell membrane by the hypertonic solution with 20–30 mM CaCl_2_ [32]. Both commercialize preparations of the full-length FVIII are manufactured in the presence of vWF in the culture medium, realized as co-expression of human vWF [10] or addition of human plasma proteins to the culture medium [9]. The presence of vWF in the culture medium diminishes the membrane-bound fraction of FVIII and protects the secreted FVIII from proteolytic degradation. It should be completely removed during the purification, thus increasing the complexity of downstream procedures and in-process controls. Commercialized process of obtaining the FVIII-BDD SQ does not involve the use of vWF in any form; and therefore the bio-analogues of FVIII-BDD SQ are expected to be completely free of vWF. Annexin V is the intracellular protein tightly binding to the acidic membranes, its use in the industrial process of FVIII production also seems non-practical. At the same time, the addition of o-phospho-L-serine to the culture medium is not expected to alter the downstream process or to increase the cost of cultivation significantly.

The proportion of the membrane-bound FVIII:C, liberated by NaCl + CaCl_2_ treatment according to [32], for 11A4H cell line was below 15% in all cases, including the culture, induced by sodium butyrate (data not shown). Addition of fetal bovine serum, containing bovine vWF, enhanced FVIII:C level in the culture medium (growth time 72 h) by about 60% (Fig. 6a). We suggest, that this modest increase of FVIII:C level is the result of FVIII protection from proteolytic breakdown by the fWF. Similarly, the addition of o-phospho-L-serine to the culture medium caused no increase in the FVIII:C level, however, it caused the decrease of VCD (Fig. 5d). We believe that the lack of phospho-L-serine effect we observed was due to the low content of phosphatidylserine in the cell membrane of 11A4H cells.

### BDD-FVIII purification and characterization of the recombinant protein

**Fig. 7:**
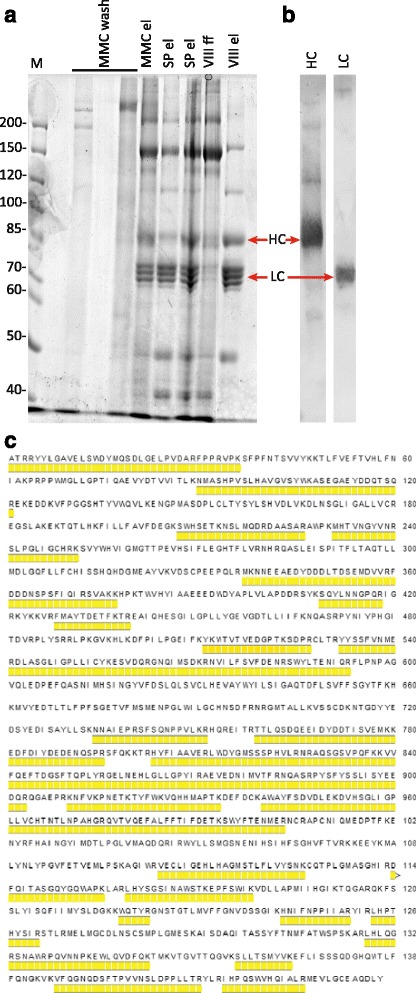
Analysis of the purified FVIII-BDD. Panel **a** - FVIII purification steps by SDS-PAGE. Panel **b** – Western blotting of purified FVIII. Panel **c** - tryptic peptides, identified by MALDI-TOF mass spectrometry. M – marker; MMC wash – wash fraction stage 1; SP el – elution fraction stage 2; VIII ff – flow-through fraction stage 3; VIII el – elution fraction stage 3. HC – heavy chain, LC – light chain. SDS-PAGE in reducing conditions, molecular weights are shown in kDa. Images were contrast enhanced to add visibility. Identified peptides are marked in *yellow*

Purification of FVIII from the culture medium was performed using the five-stage chromatography process, including the solvent/detergent viral inactivation step. SDS-PAGE analysis and Western blotting of the protein fractions collected after the first three purification stages are shown on Fig. 7a and b, the purification balance sheet is shown in Table 2. Although the visually homogeneous FVIII was obtained after the first three chromatography steps, the specific procoagulant activity of FVIII was significantly increased by subsequent anion exchange chromatography and size exclusion chromatography steps. The specific procoagulant activity of the completely purified product is comparable to the specific activity of the pharmaceutical grade FVIII-BDD SQ (7’600–13’800 IU/mg protein, according to [33]). The total yield of the FVIII protein (as measured by the procoagulant activity) was above 20%, which proves that the purification process employed is industrially applicable.

**Table 2:** FVIII purification sheet

#	Name	Resin	Volume, ml	Protein, mg/ml^a^	FVIII:C, IU/ml	Specific activity, IU/mg	Total FVIII:C, kIU	Stage yield	Overall yield
0	Clarified medium	-	320	--	36.3	-	11.6	-	-
1	Multi-mode anion exchange	Capto MMC	17	0.233	496.9	2133	8.45	73%	73%
2	Cation exchange	SP Sepharose	10	0.271	538.1	1986	5.38	64%	46%
3	Affinity	VIII Select	11	0.046	382.0	8304	4.20	78%	36%
4 + 5	Anion exchange + size exclusion	Capto Q + Superdex 75	10	0.026	284.1	10925	2.84	68%	24%

^a^1–3 protein by Bradford; stage 4 + 5 by OD_280_ nm

Tryptic peptides of the purified BDD-FVIII were analyzed by MALDI-TOF mass spectrometry. Mass spectra of tryptic peptide mixtures of the two chains of BDD-FVIII or of the single-chain FVIII form did definitely show peaks corresponding to 44% of total BDD-FVIII protein length (Fig. 7c). None of the mass-spectra, obtained in the positive mode, contained peaks corresponding to the peptide with three sulfotyrosine residues and the peptides with three fully occupied N-glycosylation sites. At the same time, we identified unmodified peptides, containing partially occupied O- glycosylation sites and peptides with T1651 and T1652 normally sulfated tyrosine residues. Thus the purified BDD-FVIII sample contains a fraction of molecules with non-sulfated tyrosine residues T1651 and T1652.

## Discussion

Specialized plasmid vector p1.1, containing non-coding areas of the *EEF1A1* gene from Chinese hamster and fragment of the terminal repeat of the Epstein-Barr virus allowed us to obtain cell lines, secreting high quantities of B-domain deleted FVIII in serum-free medium. Volumetric productivity of the clonal candidate cell line 11A4H created reached 39 IU/ml in the simple batch culture and was increased up to 75 IU/ml by induction with sodium butyrate and BHA. Previously we have created several FVIII BDD secreting cell lines using the standard CMV-based plasmid and the same transgene amplification technique [18]. These lines secreted about 0.5 IU/ml of the FVIII-BDD. The use of the specialized plasmid vector p1.1 instead of the standard vector allowed us to increase the product titer 80-fold. Other studies of the FVIII-BDD producing cell lines also report lower levels of the active protein in the culture medium; for example Chun et al. [24] reported a peak titer of 10 IU/ml of the FVIII BDD after treatment of the cell culture by the sodium propionate and around 2 IU/ml for the untreated culture. These data show that the plasmid used in the present study allows much higher secretion rates of the FVIII than standard CMV-based vectors.

After the multi-step procedure of transgene amplification, most of the genome-integrated copies of the FVIII ORF remained intact and linked to the ORF of the selection marker. This demonstrates the ability of the p1.1 vector to withstand the fragmentation of the genetic cassette upon long-term exposure to the selection agent. The selected clonal cell line showed no tendency to decrease the specific productivity for more than 70 generations without the selective pressure, demonstrating the absence of target gene silencing. The target protein, the B-domain deleted FVIII was purified to the specific activity of the pharmaceutical product with the 24% yield and characterized by mass spectrometry. The cell line created can be employed for the economical production of the bio-analogue FVIII for hemophilia A treatment.

## Conclusion

The results obtained show that a specialized plasmid vector based on the EEF1A1 gene is suitable for the development of highly productive and stable clonal cell line, secreting the large therapeutically relevant protein - blood clotting factor VIII. Additionally, we have found that the created cell line secretes factor VIII to the culture medium without significant binding of the product to the cell membrane. Novel genetic constructions for the expression of heterologous proteins combined with the optimized cultivation method allowed to obtain the secretion level of biologically active recombinant FVIII increased almost tenfold as compared with the previously published analogues.

## Additional files

Additional file 1: Table S1. Primers for Q-PCR. Sequences of primers, used for Q-PCR experiments. (RTF 76 kb)

Additional file 2: Figure S1. Cell cultures workflow diagram. Order of cell pools and lines generation, leading to final clonal cell lines, expressing FVIII. (PDF 608 kb)

Additional file 3: Raw data for figures. (ZIP 136 kb)

Additional file 4: Figure S3. Agarose gel images for Figure 3. Variant of the Figure 3C, 3D with the agarose gel images matched to the corresponding Southern blot membranes. (PDF 1 mb)

## Abbreviations

BiP: Immunoglobulin-binding protein (Grp78)
CHO cells: Chinese hamster ovary cells
DHFR: Dihydrofolate reductase (EC 1.5.1.3)
EBVTR: Concatemer of the terminal repeats from the Epstein-Barr virus
EEF1A: Chinese hamster elongation factor 1 alpha
FVIII:Ag: Concentration of the factor VIII antigen
FVIII:C: Procoagulant activity of factor VIII
IRES: Internal ribosome entry site
EMCV: Encephalomyocarditis virus
IU: International unit, procoagulant activity of FVIII from 1 ml of pooled male donors plasma
Kbp: Kilo base pairs (1000 base pairs)
MTX: Methotrexate
ORF: Open reading frame
PCR: Polymerase chain reaction
PPIB: Peptidyl-prolyl isomerase B, cyclophilin B
UPR: Unfolded protein response
VCD: Viable cell density
