# Supplementary material for: Stable high-level expression of factor VIII in Chinese hamster ovary cells in improved elongation factor-1 alpha-based system
Source: BMC Biotechnol. 2017 Mar 24;17:33. doi: 10.1186/s12896-017-0353-6 (PMC5366130; doi:10.1186/s12896-017-0353-6)
Supplement: Supplementary file 1 — Primers for Q-PCR. Sequences of primers, used for Q-PCR experiments. (RTF 76 kb) [file 12896_2017_353_MOESM1_ESM.rtf]

Supplementary Table S1. Primers for Q-PCR
Target gene	Primer name	Primer sequence (5'-3')	Target amplicon length, bp 	Calculated Tm, îÑ 	
Expression cassette copy number and target gene expression level determination1	
IRES-DHFR	RT-ID-F
RT-ID-R	GCCACAAGATCTGCCACCATG
GTAGGTCTCCGTTCTTGCCAATC	88	54.7	
FVIII-BDD	RT-BDD-F
RT-BDD-R	GACAAGAACACTGGTGATTATTAC
CGAGTTATTTCCCGTTGATGG	140	52	
HKGs expression level determination	
EiF1a13	RT-EiF1a1-F
RT-EiF1a1-R	CCGTCAGAACGCAGGTGTTG
GTGGTGGACTTGCCTGAATCTAC	116	54	
EiF32	RT-EiF3-F
RT-EiF3-R	CCACAACTTCCACCAGGATT
ATGCGGACGTAACCATCTTC	166	55	
PPIB3	RT-PPIB-F
RT-PPIB-R	GCAGGCAAAGACACCAATG
CTCCACCTTCCTCACTACATC	123	53	
BIP	RT-BIP-F
RT-BIP-R	ACTGCTTGATGTATGTCCTCTTAC
TGTTAGGGGTCGTTCACCTTC	116	52.6	
OSTC	RT-OSTC-F
RT-OSTC-R	CCTGGATTGGCTCCTGTTAG
TTACTCTGCTCTTCACTCTTCTC	82	53	
St3gal3	RT-St3gal3-F
RT-St3gal3-R	GCGGGAGTCAACAAGAGTAG
ACATGACTTTGACAAACTGACC	89	54.5	
Beta4galt1	RT-Beta4galt1-F
RT-Beta4galt1-R	TCAACATAGGCTTCCAAGAGG
AGGCAGGCTAAACCCAAAC	161	54	
ACTB2	RT-bACT-F
RT-bACT–R	GCTCTTTTCCAGCCTTCCTT
GAGCCAGAGCAGTGATCTCC	187	56	

1Primer sequences were designed to be absent in host-cell genome. The IRES-DHFR pair contains one primer to the IRES sequence of ECMV and the reverse primer to mouse dihydrofolate reductase gene region. The FVIII-BDD primers correspond to SQ linker region of the FVIII-BDD ORF, thus giving no product of appropriate length for homologous FVIII CHO gene.
2 Primers from [1]
3 genes shown to be upregulated in CHO cells producing high levels of dhfr-GFP fusion protein [2].
IRES-DHFR and FVIII-BDD– sequence of p1.1-F8 expression plasmid; EiF1a1- eukaryotic translation initiation factor 1a, EiF3- eukaryotic initiation factor 3, PPIB- peptidyl-prolyl isomerase B, BIP – immunoglobulin-binding protein (BiP, Grp78); OSTC- oligosaccharyltransferase complex subunit; St3gal – ST3 beta-galactoside alpha-2,3-sialyltransferase 3; B4gal - beta-1,4-galactosyltransferase 1, ACTB - beta actin; GADPH - Glyceraldehyde 3-phosphate dehydrogenase

References:
1.	Bahr SM, Borgschulte T, Kayser KJ, Lin N: Using microarray technology to select housekeeping genes in Chinese hamster ovary cells. Biotechnol Bioeng 2009, 104(5):1041-1046.
2.	Nissom PM, Sanny A, Kok YJ, Hiang YT, Chuah SH, Shing TK, Lee YY, Wong KT, Hu WS, Sim MY et al: Transcriptome and proteome profiling to understanding the biology of high productivity CHO cells. Mol Biotechnol 2006, 34(2):125-140.
