# Supplementary material for: Stable high-level expression of factor VIII in Chinese hamster ovary cells in improved elongation factor-1 alpha-based system
Source: BMC Biotechnol. 2017 Mar 24;17:33. doi: 10.1186/s12896-017-0353-6 (PMC5366130; doi:10.1186/s12896-017-0353-6)
Supplement: Supplementary file 2 — Cell cultures workflow diagram. Order of cell pools and lines generation, leading to final clonal cell lines, expressing FVIII. (PDF 608 kb) [file 12896_2017_353_MOESM2_ESM.pdf]

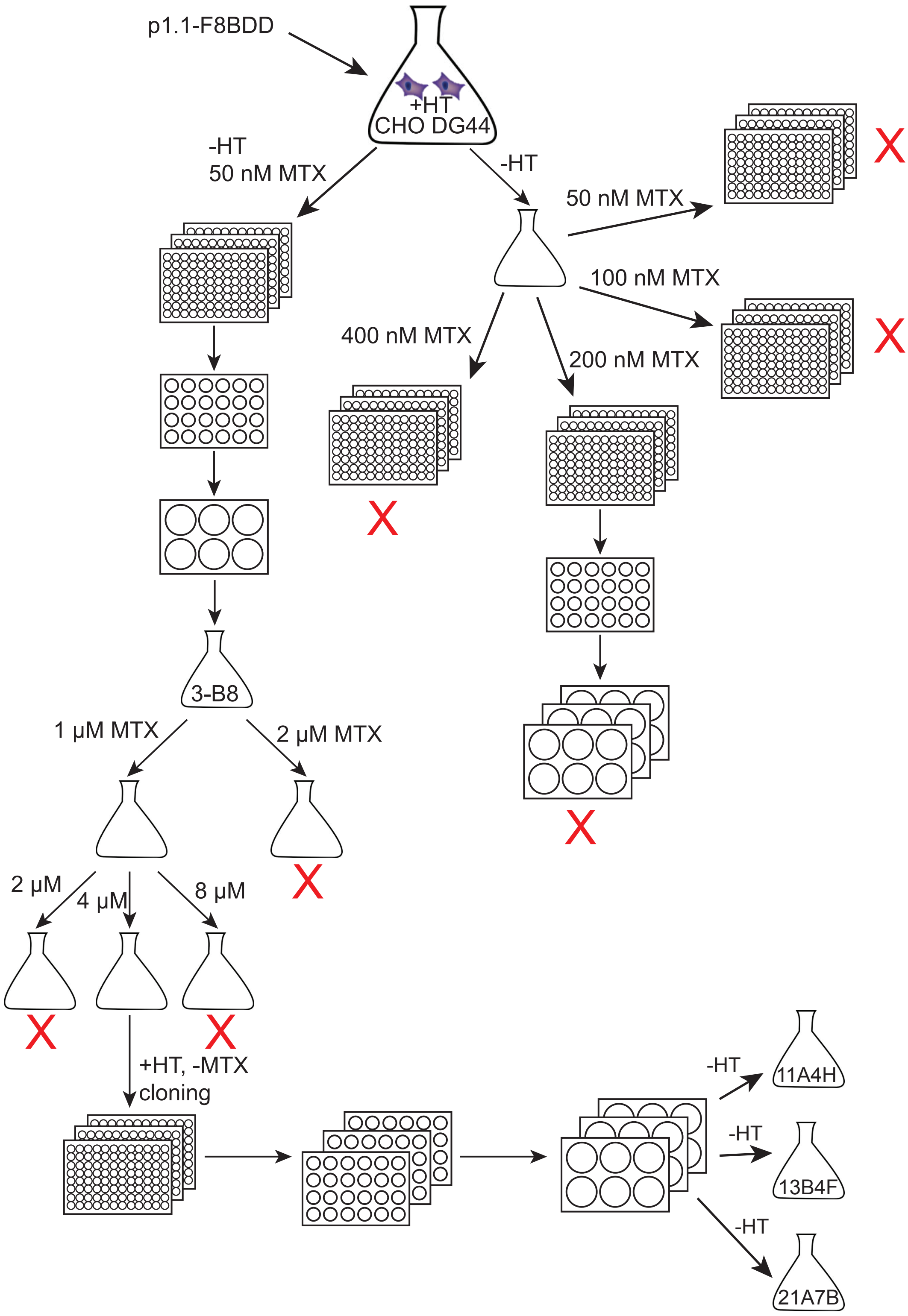

Supplementary Figure S1. Cell cultures workflow diagram. Red cross means culture termination. Cultivation in flasks and 6-well plates with shaking, in 96-w and 24-w plates without shaking and without Pluronic-F68.
