# Supplementary figures and images for: Stable high-level expression of factor VIII in Chinese hamster ovary cells in improved elongation factor-1 alpha-based system
Source: BMC Biotechnol. 2017 Mar 24;17:33. doi: 10.1186/s12896-017-0353-6 (PMC5366130; doi:10.1186/s12896-017-0353-6)

# Agarose gel images for Figure 3

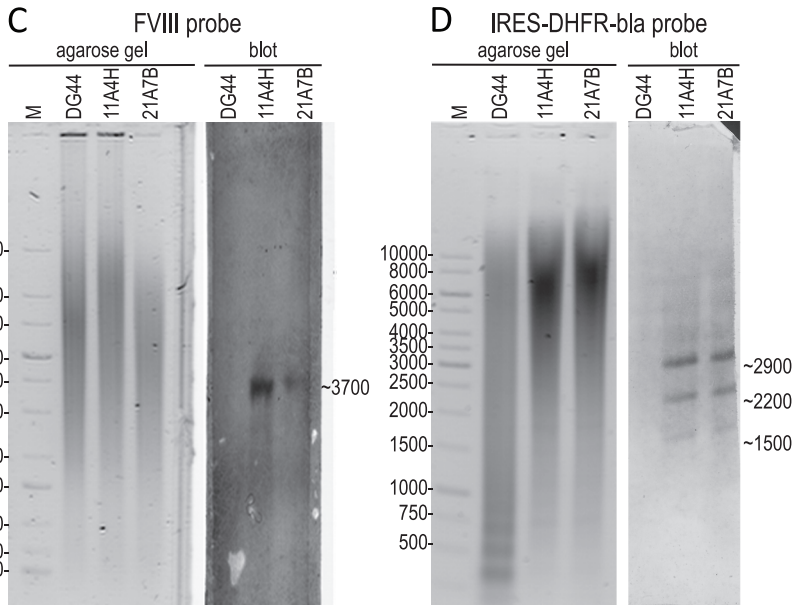

Supplement: Supplementary file 4 — Agarose gel images for Figure 3. Variant of the Figure 3C, 3D with the agarose gel images matched to the corresponding Southern blot membranes. (PDF 1 mb) [file 12896_2017_353_MOESM4_ESM.pdf]
